# Conditional Stat2 Knockout Mice as a Platform for Modeling Human Diseases

**DOI:** 10.3390/immuno6010007

**Published:** 2026-01-12

**Authors:** Tess Cremers, Nataliya Miz, Alexandra Afanassiev, Ling Yang, Kevin P. Kotredes, Ana M. Gamero

**Affiliations:** 1 Department of General Surgery, Cooper University Hospital, Camden, NJ 08103, USA; 2 Department of Medical Genetics and Molecular Biochemistry, Lewis Katz School of Medicine, Temple University, Philadelphia, PA 19140, USA; 3 The Jackson Laboratory, Bar Harbor, ME 04609, USA

**Keywords:** STAT2, type I interferon, conditional knockout, Cre deletion, tumor, antiviral, tissue-specific deletion

## Abstract

Signal transducer and activator of transcription 2 (STAT2) is a key component of the type I interferon (IFN-I/III) signaling pathway, which is pivotal in host defense against cancer and viral infections and in shaping immune responses. Building on our previously reported conditional *Stat2 knockout (KO)* mouse, we expand its utility by validating additional tissue-specific models and exploring novel functional contexts. Mice carrying loxP-flanked *Stat2* alleles were crossed with *CMV-Cre, Cdx2-Cre* or *CD11c-Cre* mice. Deletion of STAT2 was validated by PCR genotyping and western blotting in the relevant tissues. To confirm defective IFN-I signaling with STAT2 deletion, IFN-β stimulation of splenocytes from CMV-Cre *Stat2 KO* mice showed a lack of induction of canonical IFN-I target genes, confirming functional disruption of the pathway. In vivo, global *Stat2* deletion significantly impaired the antitumor efficacy of IFN-β treatment. Similarly, lung fibroblasts isolated from globally deleted *Stat2 KO* mice showed defective antiviral responses to IFN-β. Tissue-specific Cre models demonstrated selective ablation of STAT2 in target compartments without affecting its expression in non-target tissues. Together, these studies expand our published conditional *Stat2 KO* findings and highlight the value of this model as a versatile platform for dissecting STAT2-dependent signaling pathways in a tissue- and disease-specific manner.

## 1. Introduction

Signal transducer and activator of transcription 2 (STAT2) is a central downstream effector of type I and type III interferon (IFN-I/III) signaling and a key regulator of antiviral and antitumor immunity and immune homeostasis [1–3]. IFN-I/III-induced activation of tyrosine kinases JAK1 and TYK2 results in the phosphorylation of STAT2 and STAT1, enabling their heterodimerization and association with IRF9 to form the interferon-stimulated gene factor 3 (ISGF3) transcriptional complex and transcription of interferon-stimulated genes (ISGs) [4]. Through this canonical pathway, STAT2 has long been regarded as operating strictly within the IFN-I/III signaling axis.

Recent findings, however, reveal that this canonical view does not fully capture STAT2 biology. STAT2 can participate in signaling programs independent of IFN stimulation or even in the absence of STAT1, influencing metabolic pathways, inflammatory regulation, and tissue-specific immune function [5]. Importantly, phenotypic differences between *Stat1* and *Stat2 knockout (KO)* mice underscore that STAT2 performs unique, non-redundant roles that cannot be inferred from STAT1 loss alone [6,7]. These observations highlight the need to interrogate STAT2 directly within defined cellular and tissue contexts.

Despite increasing recognition of STAT2 functions beyond canonical IFN-I/III signaling, mechanistic dissection of STAT2 activity in vivo has been constrained by the absence of a conditional genetic model. Although global *Stat2 KO* mice have been instrumental in defining defects in IFN-I signaling, they do not allow disentangling between systemic and tissue-restricted effects, nor do they permit evaluation of cell-intrinsic STAT2 functions within distinct specialized immune or stromal compartments [8,9]. As STAT2 becomes increasingly implicated in tumor biology and context-dependent inflammatory processes, the availability of a flexible genetic system for targeted Stat2 deletion has become critical. Notably, we previously reported targeted deletion of Stat2 in conventional dendritic cells (cDCs), which revealed a critical cell-intrinsic role for STAT2 in mediating antitumor immunity in vivo [10]. Similarly, another group reported the deleterious cell-intrinsic effect of STAT2 in pancreatitis [11]. This study demonstrated that tissue-restricted Stat2 ablation can uncover biologically important functions that are masked in global knockout models, further highlighting the need for versatile conditional approaches.

Building on our previously reported conditional *Stat2 KO* model, here, we validate additional tissue-specific Cre lines and demonstrate its versatility for studying STAT2-dependent signaling in both canonical and non-canonical pathways in vivo. This model enables investigation of STAT2 functions in diverse disease contexts, including cancer, viral infection, and tissue-specific immune regulation.

## 2. Materials and Methods

### 2.1. Generation of Stat2 Floxed Mice

*Stat2* conditional knockout mice *(Stat2*^*fl/fl*^*)* were generated with Biocytogen (Waltham, MA, USA) using a gene-targeting strategy in which exons 5–8 of the *Stat2* locus were flanked by loxP sites. The targeting construct was introduced into C57BL/6 (B6) embryonic stem cells by electroporation with the distal loxP site linked to a neomycin-resistant cassette flanked by FRT sites. Correct genomic integration was verified by Southern blot analysis. The neomycin resistance cassette was subsequently excised by crossing with deleter mice carrying Flp recombinase. F1 heterozygous for the floxed *Stat2* allele were intercrossed to obtain homozygous Stat2^*fl/fl*^ mice. All animal experiments were approved by the Temple University Institutional Animal Care and Use Committee. Wild-type (WT) and *Stat2 KO* mice, previously backcrossed onto the B6 genetic background [12], were bred and maintained in our pathogen-free animal facility. The following mouse strains on the B6 background were purchased from The Jackson Laboratory: *FLPe deleter* (Strain#: 009086), *CMV-Cre* (Strain#: 006054), *CD11c-Cre* (Strain#: 008068) and *Cdx2-Cre* (Strain#: 009350). Global *Stat2* deletion was achieved by crossing Stat2^*fl/fl*^ mice with *CMV-Cre* mice. Targeted deletion of *Stat2* in cDCs and colonic epithelial cells was accomplished by crossing Stat2^*fl/fl*^ mice with *CD11c-Cre+ or Cdx2-Cre* mouse strains, respectively. Deletion of *Stat2* was verified by genotyping using primers in Appendix A; Table A2.

### 2.2. qRT-PCR Analysis

Total RNA was isolated from individual mouse tissues stored in RNA-later stabilization solution (cat#AM7020; Invitrogen, Carlsbad, CA, USA) using Trizol^®^ Reagent (Invitrogen, Carlsbad, CA, USA). Contaminating DNA in RNA samples was removed with a DNA-free removal kit (cat#AM1906; Invitrogen, Carlsbad, CA, USA). ISG expression in freshly isolated splenocytes treated with or without recombinant murine IFN-β (1000 U/mL) at 37 °C for 6 h was determined by qRT-PCR. RT-PCR was performed as a two-step process using High-Capacity cDNA Reverse Transcription (Applied Biosystems; Foster City, CA, USA) and SYBR Green (Bioland Scientific LLC, Los Angeles, CA, USA). Each cDNA sample was run in triplicate using the Step One Plus Real Time PCR system (Applied Biosystems). Primer sequences were obtained from Harvard PrimerBank [13] or from the published literature (Appendix A; Table A2). Results were analyzed using the comparative Δ CT method. Data were normalized to Gapdh. Relative Stat2 gene expression in *Stat2*^*Δ/Δ*^ or *Stat2 KO* tissues was calculated relative to WT control cells. Relative ISG expression in IFN-β treated cells was calculated relative to its corresponding untreated cells.

### 2.3. Tumor Cell Lines, Antibodies and Cytokines

Murine B16-F1 melanoma cells were cultured in DMEM medium (Mediatech, Inc; Herndon, VA, USA) and supplemented with 5% heat-inactivated fetal bovine serum (FBS), 2 mM L-glutamine, 1 mM sodium pyruvate, penicillin (100 U/mL) and streptomycin (100 μg/mL) (Invitrogen Corp; Carlsbad, CA, USA) at 37 °C in 5% CO2. Murine EL-4 lymphoma cells were cultured in DMEM medium containing 10% heat-inactivated horse serum, L-glutamine and sodium pyruvate. Murine IFN-β was generously provided by Biogen, Idec. GM-CSF was purchased from BD Biosciences, San Jose, CA (cat# 554586). Antibodies against STAT1 (Cat#10144–2-AP), STAT2 (cat#51075–2-AP), β-Actin (cat#66009–1-Ig), HRP-conjugated anti-mouse IgG (cat#SA00001–1) and anti-rabbit-IgG (cat#SA00001–2) were purchased from Proteintech, Rosemont, IL, USA.

### 2.4. Tumor Transplantation

C57BL/6 mice (6–8 weeks old) received a single subcutaneous (s.c.) injection in the dorsal flank of either 1 × 10^6^ B16-F1 melanoma cells or 3 × 10^5^ EL4 lymphoma cells suspended in 200 μL of endotoxin-free 0.9% saline solution. Tumor measurements started at day 7 using a digital caliper. Tumor volume was determined with the formula V = a^2^b, where a is the shorter diameter and b is the longer diameter of the tumor. The study was terminated when the tumors reached a diameter of 20 mm. No unexpected deaths occurred during the study.

### 2.5. Western Blot Analysis

Cells and tissues were lysed as previously described [12]. Protein lysates were resolved on precast SurePAGE 4–12% gradient gels (GenScript, Piscataway, NJ, USA) and transferred to polyvinylidene difluoride membranes. Membranes were blocked with Casein Blocker in TBS (Bio-Rad, Hercules, CA, USA) and incubated with the appropriate primary antibodies followed by HRP-conjugated secondary antibodies in TBS-T + 3% BSA. Protein signals were detected using enhanced chemiluminescence reagent (Cat# 1705060; Bio-Rad) and visualized with a Bio-Rad ChemiDoc imaging system. β-actin served as an internal loading control.

### 2.6. Vesicular Stomatitis Virus (VSV) Infection

Lung fibroblasts of varying genotypes seeded in 12-well plates were left untreated or pretreated with 100 U/mL of murine IFN-β for 24 h. Cells were then infected with vesicular stomatitis virus (VSV) with a GFP-expressing gene [14]. VSV was added to cells at a multiplicity of infection of 0.01 for WT, *Stat2 KO*, *Stat2*^*fl/fl*^ and *Stat2*^*Δ*/*Δ*^ fibroblasts under serum-free medium conditions for 1 h at 37 °C. Cells were washed twice with PBS. Complete DMEM was then re-added. Cells were imaged using a Nikon inverted fluorescent microscope after 24 h.

### 2.7. Lung Fibroblasts Isolation

Lung tissue fragments were extracted from 3 to 5-week-old mice and transferred into a tissue culture dish according to an established protocol [15]. The fragments were cut into 1 mm pieces, washed with PBS and placed into a beaker containing Collagenase II (2 mg/mL) and DNase I (100 μg/mL). The cells were incubated for 1 h at 37 °C. The solution was pipetted to break down clumps and transferred to a 50 mL tube where FBS was added to stop digestion. Cells were spun down and resuspended in complete media (DMEM contained 10% FBS and 1% penicillin–streptomycin). Cells were transferred to a tissue dish and incubated at 37 °C overnight. The plates were monitored for changes in media color and washed to remove non-viable cells. The cells were incubated for 7–14 days before use.

### 2.8. Bone Marrow–Derived Conventional Dendritic Cells

Bone marrow–derived DCs were generated, as previously described [10], from different mouse genotypes. Briefly, bone marrow precursors were flushed from the femurs and tibias of mice and then seeded at 5 × 10^5^/well in complete IMDM (Mediatech, Manassas, VA, USA) (10% FBS, penicillin/streptomycin, gentamicin and 2-ME) (Life Technologies, Grand Island, NY, USA) and enriched with 3.3 ng/mL GM-CSF in 48-well plates or at 10^6^/well in 24-well plates. Half medium was added on day 2, and half was replaced on day 5 and on each subsequent day until the culture was used for Western blot analysis.

### 2.9. Statistical Analysis

Prism software (Version 8, GraphPad, San Diego, CA, USA) was used for statistical analysis. In vitro results were analyzed using the Student’s *t*-test to assess significance. In comparing multiple parameters, two-tailed one-way ANOVA followed by Dunn’s multiple comparison test was applied. In vivo data were analyzed using the Mann–Whitney U test. Values of *p* ≤ 0.05 were considered statistically significant. Experiments were repeated 2 to 4 times. All data are presented as mean ± SEM.

## 3. Results

### 3.1. Targeting Strategy for Generating Conditional Stat2 KO Mice

**Figure 1.**
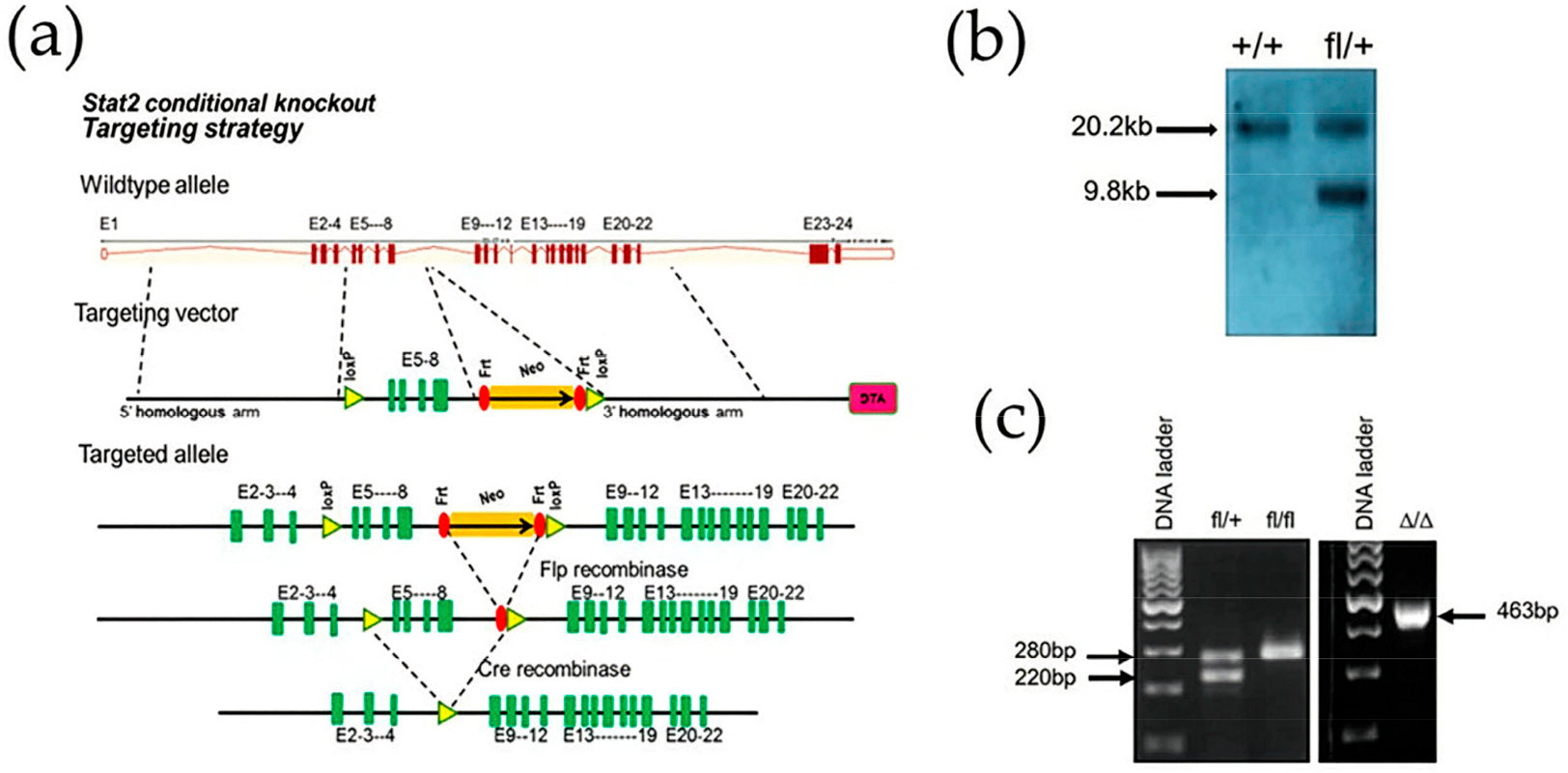
Generation of conditional *Stat2 KO* mice. (**a**) Targeting strategy used to construct *Stat2* ^*fl/fl*^ mice with exons 5 through 8 flanked with loxP sites. E represents exons with their corresponding number. Brown boxes depict exons in wild type allele and green boxes depict exons after successful integration of targeting vector. (**b**) Confirmation of loxP sites integration in the *Stat2* gene by Southern blot analysis (9.8 kb DNA fragment). (**c**) Genotyping of mouse-tail DNA by PCR confirms deletion of *Stat2* floxed allele after breeding with CMV-Cre mouse.

We generated a conditional *Stat2*^*fl/fl*^ mouse to investigate the specific contribution of cell-autonomous STAT2 function in IFN signaling and cancer. The mouse *Stat2* gene contains 24 exons, spans approximately 22 kilobases and is located on chromosome 10 (forward strand). Exons 5–8 were selected because their removal eliminates a critical protein domain of STAT2, resulting in a frameshift mutation and a non-functional protein. Global Cre-mediated deletion of the floxed Stat2 locus (*Stat2*^*Δ/Δ*^) was achieved by crossing *Stat2*^*fl/fl*^ mice with those expressing ubiquitous CMV-Cre-recombinase. This particular Cre strain was selected to ensure efficient global deletion of Stat2 in the *Stat2*^*fl/fl*^ background. The resulting mice were fertile and viable, consistent with the previously reported phenotype of conventional *Stat2 KO* mice [8]. Targeted deletion removed exons 5–8, and Cre-mediated recombination introduced a frameshift predicted to generate a truncated protein of 153 amino acids (Figure 1a,b). Efficient *Stat2* deletion was confirmed by genotyping, which produced a 436 bp band in contrast to the 220 bp and 280 bp bands observed in wild-type (WT) and *Stat2*^*fl/fl*^ mice, respectively (Figure 1c)

### 3.2. Impaired IFN-I Signaling in Stat2-Deleted Mice

**Figure 2.**
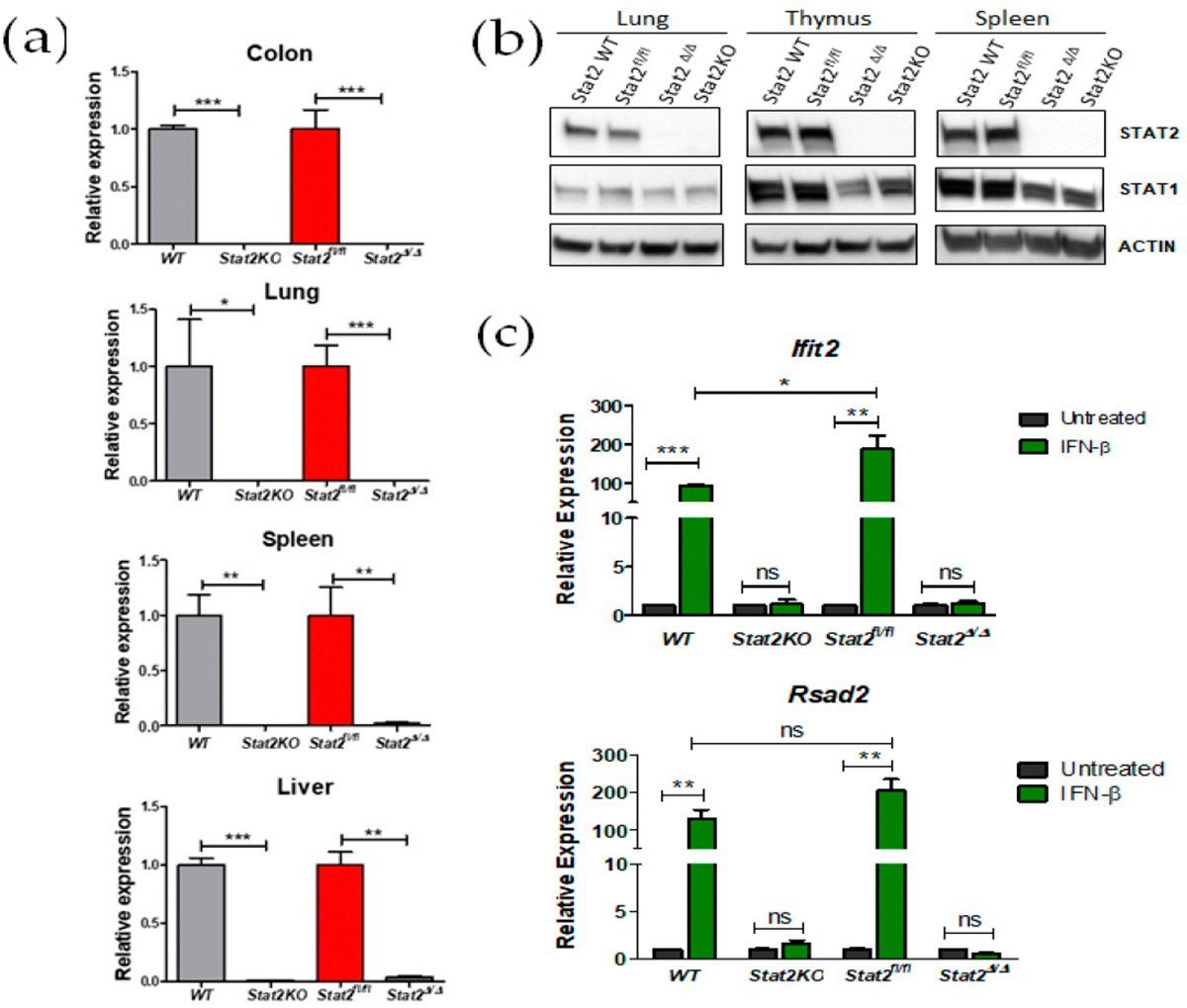
Impaired IFN-I signaling after conditional deletion of Stat2 by CMV-Cre—mediated recombination. (**a**) Loss of Stat2 gene expression validated in four different organs by qRT-PCR analysis. (**b**) Western blot analyses performed on several tissues confirm global Stat2 deletion (*Stat2*^*Δ*/*Δ*^) by ubiquitous CMV-Cre recombinase. (**c**) Splenocytes of the indicated genotypes were left untreated or treated with IFN-β for 6 h, and ISG expression was determined by qRT-PCR. * *p* ≤ 0.05; ** *p* ≤ 0.01; *** *p* ≤ 0.001. Data are presented as SEM of three independent experiments. n.s; not significant.

We confirmed that loss of Stat2 mRNA across multiple organs (colon, lung, spleen and liver) in *Stat2*^Δ/Δ^; *CMV-Cre* mice, and the extent of this loss was comparable to that observed in conventional *Stat2 KO* mice (Figure 2a). Similarly, Stat2 protein expression was also absent (Figure 2b; Figure S1). In addition, reduced levels of STAT1 protein were tissue-dependent in global *Stat2*^*Δ/Δ*^ mice, faithfully recapitulating a known feature of *Stat2 KO* mice [8]. As expected, induction of IFN-I target genes (*Rsad2 and Ifit2*) was markedly impaired in both Stat2^Δ/Δ^ and conventional *Stat2 KO* mice (Figure 2c). Altogether, these data confirm that the floxed Stat2 allele was efficiently deleted, resulting in defective IFN-I signaling.

### 3.3. Tumor Growth Is Accelerated in Stat2-Deleted Mice

**Figure 3.**
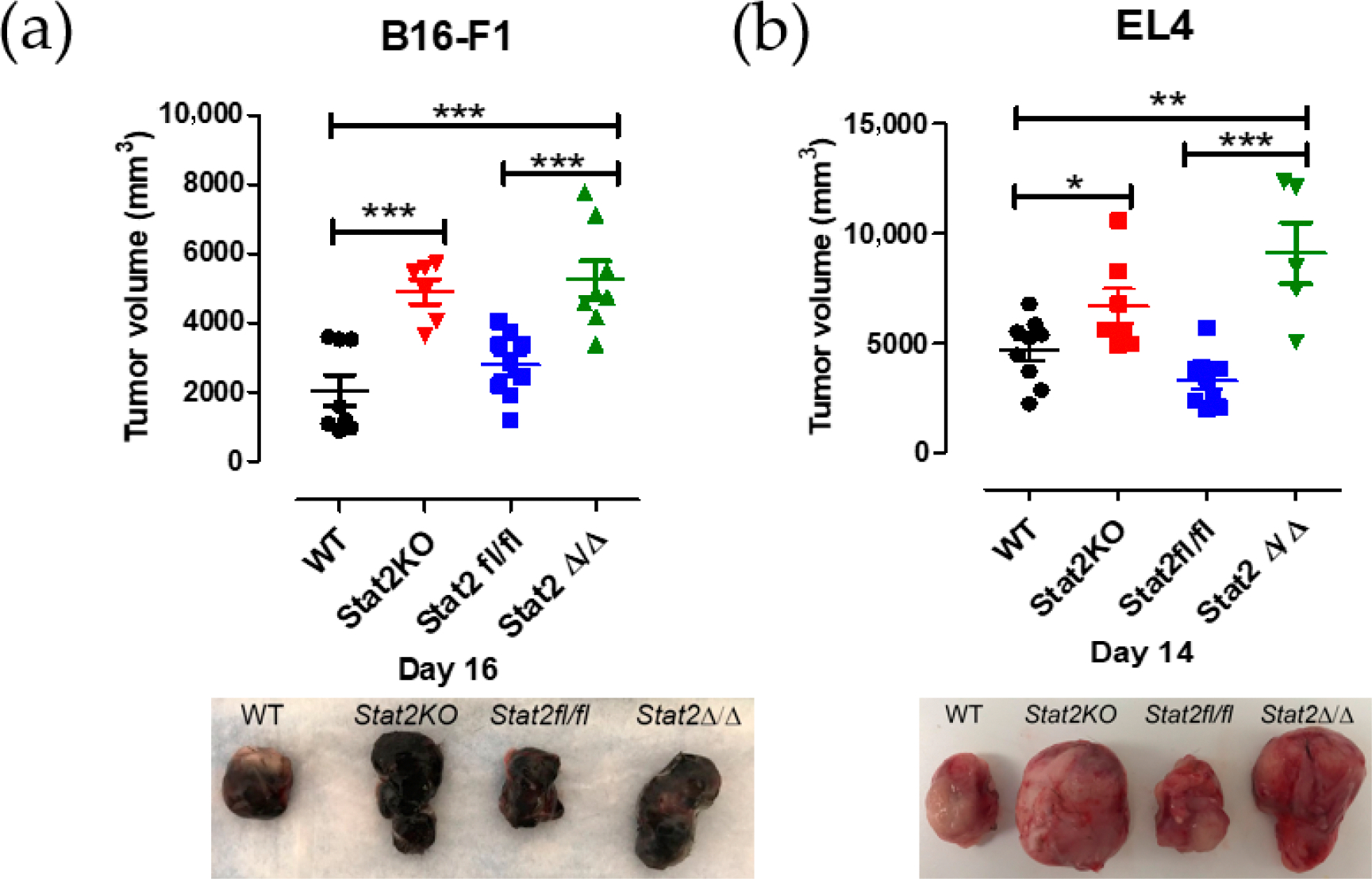
*Stat2*^*Δ*/*Δ*^ mice show enhanced tumor growth. (**a**) B16-F1 or (**b**) EL4 tumor cells were injected subcutaneously in WT, *Stat2KO*, *Stat2fl/fl* and *Stat2*^*Δ/Δ*^. Values are shown as mean tumor volume determined over 20 days. Representative images of individual tumors are shown. *, *p* ≤ 0.05; **, *p* ≤ 0.01; and ***, *p* ≤ 0.001. *n* = 6–8 animals/group.

We previously reported that, in a syngeneic tumor transplantation model, *Stat2 KO* mice developed larger tumors than WT mice [12]. We selected the B16-F1 and EL4 cell lines because they are reliably tumorigenic in vivo and reproducibly form measurable tumors within 2–3 weeks. Consistent with these findings, *Stat2*^*Δ*/*Δ*^ mice that received a subcutaneous injection of either B16-F1 or EL4 tumor cells formed progressively larger tumors compared with WT and *Stat2*^*fl/fl*^ mice (Figure 3a,b). Together, these results confirm that ubiquitous Cre-mediated Stat2 deletion in *Stat2*^*fl/fl*^ mice was effective and recapitulates the tumor-promoting phenotype observed in *Stat2 KO* mice, highlighting the importance of STAT2 in the hostile tumor microenvironment.

### 3.4. Stat2 Deletion Compromises IFN-I–Mediated Antiviral Protection in Lung Fibroblasts

**Figure 4.**
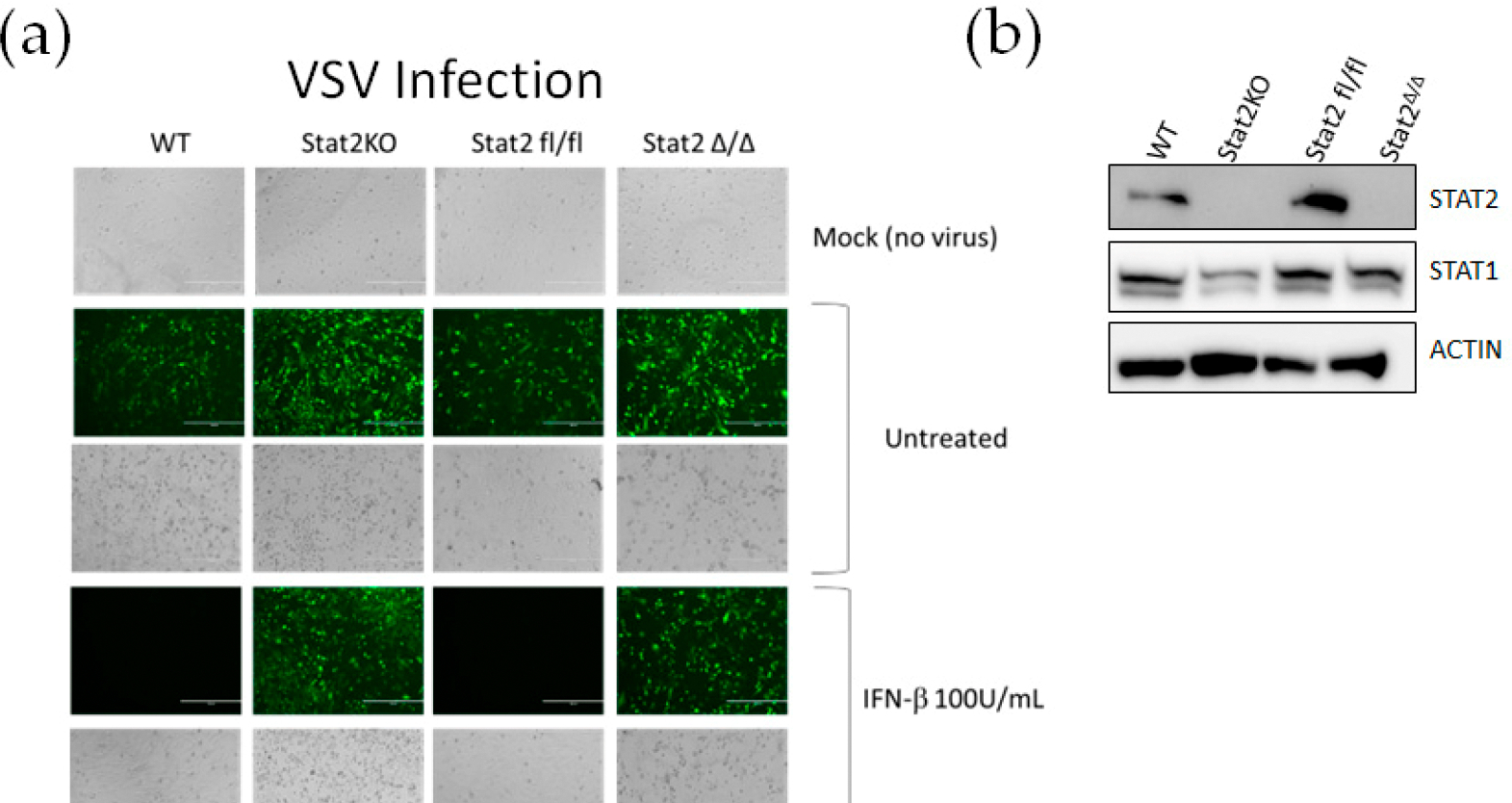
*Stat2*^*Δ*/*Δ*^ fibroblasts have impaired antiviral response to type I IFNs. (**a**) Lung fibroblasts derived from the indicated genotypes were pretreated with IFN-β for 24 h. Cells were then infected with VSV-GFP (green) at an MOI of 0.01 and imaged after 24 h. Representative bright field and fluorescent images are shown. (**b**) Western blot analysis shows STAT2 expression in fibroblasts of indicated genotypes. Representative images are shown of n = 2.

STAT2 plays a critical role in mediating the antiviral effects of IFN-I and IFN-III, as documented in individuals born with a STAT2 deficiency [16]. To evaluate the IFN-I induced antiviral response, we used vesicular stomatitis virus expressing GFP (VSV-GFP) as a reporter of infection. Lung fibroblasts isolated from WT, *Stat2 KO*, *Stat2*^*fl/fl*^ and *Stat2*^*Δ/Δ*^ mice were left untreated or pretreated overnight with 100 U/mL IFN-β and subsequently infected with VSV-GFP (Figure 4a; Figure S2). Loss of Stat2 protein in *Stat2KO* and *Stat2*^*Δ/Δ*^ fibroblasts was confirmed by Western blot analysis prior to viral infection (Figure 4b). Compared with WT and *Stat2*^*fl/fl*^ fibroblasts, both untreated *Stat2 KO* and *Stat2*^*Δ/Δ*^ exhibited a markedly higher level of infection. As expected, pretreatment with IFN-β conferred antiviral protection only in WT and *Stat2* ^*fl/fl*^ fibroblasts, whereas Stat2-deficient fibroblasts remained fully susceptible to VSV.

### 3.5. Conditional Stat2 Allele Enables Efficient Cre-Restricted Deletion

**Figure 5.**
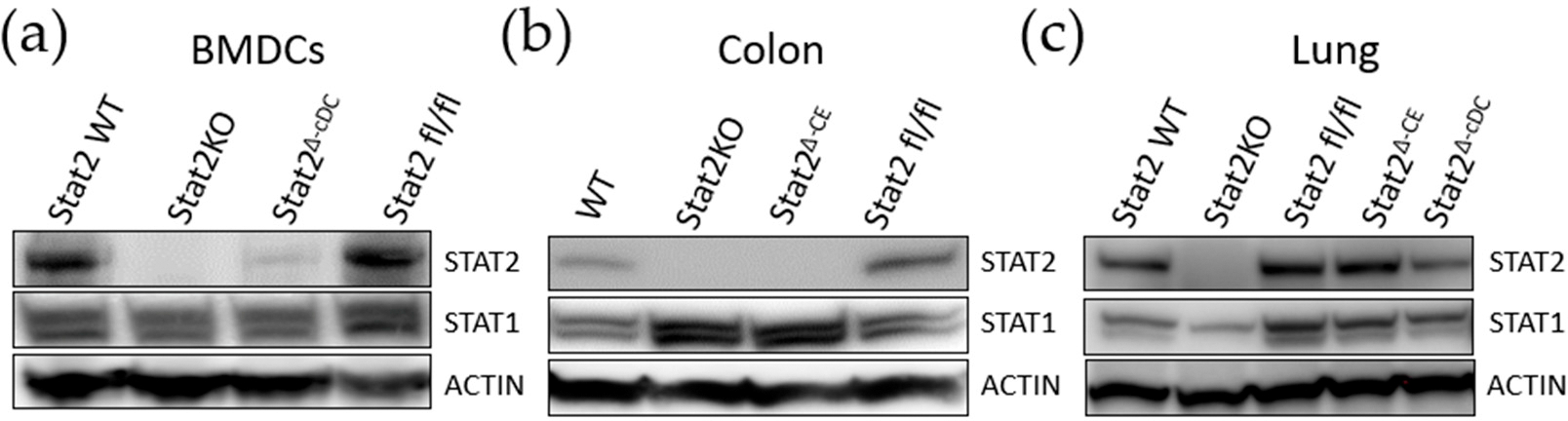
Efficient targeted deletion of Stat2 in mouse tissues. Expression of STAT2 and STAT1 was analyzed in various tissues by Western blot analysis. (**a**) Bone marrow–derived cDCs generated from *Stat2* ^*fl/fl*^ mice crossed with CD11c-Cre mice (*Stat2*^*Δ-cDC*^). (**b**) Colons from *Stat2* ^*fl/fl*^ mice crossed with Cdx2-Cre mice (*Stat2*^*Δ-CE*^). (**c**) Lungs from *Stat2*^*Δ-cDC*^ and *Stat2*^*Δ-CE*^ mice were included for specificity of targeted deletion. Wild-type (WT) and *Stat2KO* mice served as positive and negative controls, respectively. ACTIN was used as an internal protein loading control.

To further validate the specificity and efficiency of our conditional *Stat2 KO* mouse, we crossed *Stat2*^*fl/fl*^ mice with *CD11c-Cre* mice to delete STAT2 in conventional dendritic cells (*Stat2*^*Δ*-*cDC*^) and with *Cdx2-Cre* mice to delete STAT2 in colonic epithelial cells (*Stat2*^*Δ-CE*^). We assessed STAT1 and STAT2 protein expression by Western blot across multiple tissues, including bone marrow-derived DCs (BM-DCs), the colon and the lung. Robust expression of both proteins was observed in WT and *Stat2* ^*fl/fl*^ mice, whereas *Stat2 KO* tissues showed loss of STAT2 expression and marked reduction in STAT1 levels only in the lungs (Figures 5a–c and S3). No marked differences in STAT1 levels were noted in BM-DCs. STAT2 expression was absent in cDCs of Stat2^*Δc-DC*^ and in colons of *Stat2*^*Δ-CE*^ mice, consistent with the specific activity of these Cre drivers. Unexpectedly, we found that colons from *Stat2 KO* and *Stat2*^*Δ-CE*^ mice had increased STAT1 expression, whereas STAT2 levels in the lungs of Stat2^*Δ-cDC*^ and *Stat2*^*Δ-CE*^ mice remained unaffected. To our knowledge, this is the first time increased STAT1 levels in the absence of STAT2 have been observed in colons. Together, these findings demonstrate that targeted Stat2 deletion is efficient in the global knockout setting and specific to Cre-expressing lineages (Figure 5). More importantly, targeted STAT2 deletion can affect STAT1 levels in a tissue-dependent manner.

## 4. Discussion

Our study expands on the characterization of a conditional *Stat2 KO* mouse as a versatile platform to investigate STAT2 function in a tissue-specific and cell-intrinsic manner. Consistent with prior work, global deletion of Stat2 reproduced canonical phenotypes—including impaired IFN-I signaling, reduced STAT1 expression, enhanced tumor growth and defective antiviral responses—confirming that the floxed allele faithfully disrupts STAT2 function across tissues [8,10].

Building on earlier findings, we previously demonstrated that global and targeted loss of STAT2 in cDCs compromises the antitumor effects of IFN-I, and that global Stat2 deficiency further accelerates tumor growth [10]. Here, we show impaired IFN-I–mediated antiviral responses in Stat2-deleted fibroblasts; an established antiviral function of STAT2 [17]. These complementary data reinforce that the conditional allele produces biologically meaningful outcomes in distinct cellular contexts and support its utility for dissecting STAT2-dependent pathways.

A key strength of this model is its capacity for precise, Cre-restricted deletion, as demonstrated here in cDCs and colonic epithelial cells. This specificity preserves STAT2 expression in non-target tissues and overcomes a major limitation of conventional Stat2 KO models that cannot separate systemic from cell-intrinsic functions in vivo [5]. Importantly, conventional studies often rely on isolating tissues or cells from knockout animals for in vitro experiments. While informative, these approaches cannot fully capture tissue- and cell-specific interactions in vivo. The conditional *Stat2 KO* model addresses this limitation by allowing targeted deletion directly within the native physiological environment.

Beyond canonical IFN-I/III signaling, STAT2 also participates in non-canonical pathways regulating cellular metabolism, inflammation and tissue-specific immunity, some of which occur independently of STAT1 [18–20]. The conditional *STAT2 KO* model enables investigation of these roles in specific cell compartments, providing a framework to disentangle tissue- and cell-type-specific functions. For example, a wide range of tissue-specific Cre lines can be applied in gut inflammation or metabolic dysfunction models to clarify how STAT2 regulates context-dependent inflammatory responses within organs or specific cell populations. By facilitating such precise mechanistic studies, this platform offers a versatile tool to define both canonical and non-canonical STAT2 functions in tumor biology, cell metabolism, antiviral defense and immune regulation.

In summary, this conditional *Stat2 KO* model provides a powerful and flexible genetic platform to interrogate STAT2 function in vivo across tissues and disease settings, enabling mechanistic insight that is not achievable with conventional KO models.

## 5. Conclusions

Our conditional *Stat2 KO* mouse allows both global and cell–type–specific deletion of STAT2. Global deletion recapitulated known knockout phenotypes, including impaired IFN-I signaling, reduced STAT1 protein expression, enhanced tumor growth and impaired antiviral protection. Cell-type–specific deletion was precise and restricted to Cre-expressing lineages, demonstrating the fidelity of targeted ablation. These results establish the conditional *Stat2 KO* model as a powerful, versatile platform for dissecting STAT2’s tissue-specific roles in immunity, tumor biology, metabolism and antiviral defense.

## Supplementary Material

supplementary materials

**Supplementary Materials:** The following supporting information can be downloaded at https://www.mdpi.com/article/10.3390/immuno6010007/s1, Figure S1:Integration of *Stat2* targeting vector and validation by genotyping. Figure S2: Levels of STAT1 and STAT2 after in vivo CMV-Cre recombination. Figure S3: Levels of STAT1 and STAT2 in isolated lung fibroblasts in different genotypes. Figure S4: Level of STAT1 and STAT2 after targeted deletion in cDCs and colonic epithelium.

## Data Availability

The data presented in this study are fully available.

## Appendix A

**Table A1.** Primer sequences for genotyping.

***Stat2^Δ/Δ^* (463 bp)**
Forward: 5′-TGTCTCAGACCAGGATCTCCTCCAC-3′
Reverse: 5′-ATGCGGCAAACCCCACTGTAAATAG-3′
***Stat2WT* (227 bp); *Stat2^fl/fl^* (285 bp)**
Forward: 5′-TAATCCTAGCATTCTGGGCTGCA-3′
Reverse: 5′- GTTCCGAGTGTGTTTGAACTCTGA-3′

**Table A2.** qPCR primer sequences.

Gene	Forward	Reverse
Rsad2	TGCTGGCTGAGAATAGCATTAGG	GCTGAGTGCTGTTCCCATCT
Ifit2	AGTACAACGAGTAAGGAGTCACT	AGGCCAGTATGTTGCACATGG
Stat2	CTGAAGGACGAACAGGATGTC	CAGGGTGGTTAATCGGCCAA
Gapdh	TGTAGACCATGTAGTTGAGGTCA	AGGTCGGTGTGAACGGATTTG

## Abbreviations

The following abbreviations are used in this manuscript:

STAT2: Signal transducer and activator of transcription 2
CE: Colonic epithelium
cDCs: Conventional dendritic cells
FL: Floxed
IFN: Interferon
GFP: Green fluorescent protein
ISGF3: Interferon-stimulated gene factor 3
HRP: Horseradish peroxidase
KO: Knockout
PCR: Polymerase chain reaction
VSV: Vesicular stomatitis virus
WT: Wild type
