# Supplementary figures and images for: Conditional Stat2 Knockout Mice as a Platform for Modeling Human Diseases
Source: Immuno. Author manuscript; Available in PMC 2026 Mar 28. (PMC13026504; doi:10.3390/immuno6010007)

Figure S1a (Figure 1b)

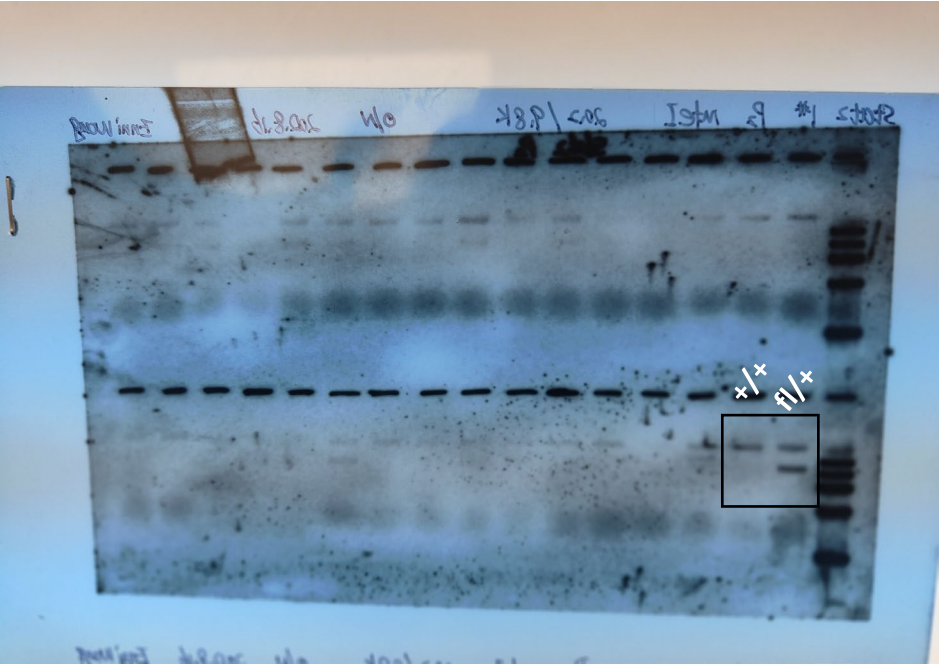

Figure S2 (Figure 2b)

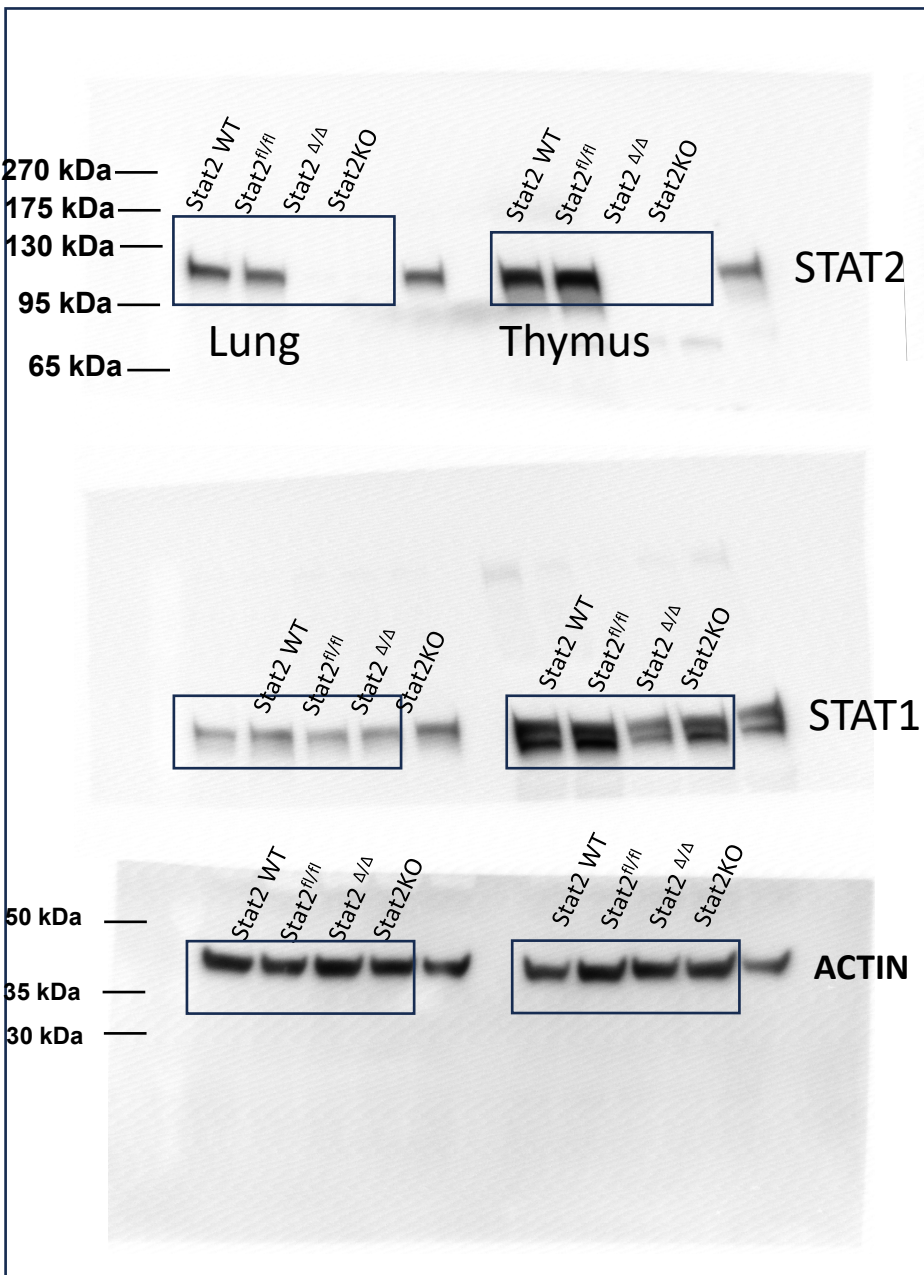

Spleen

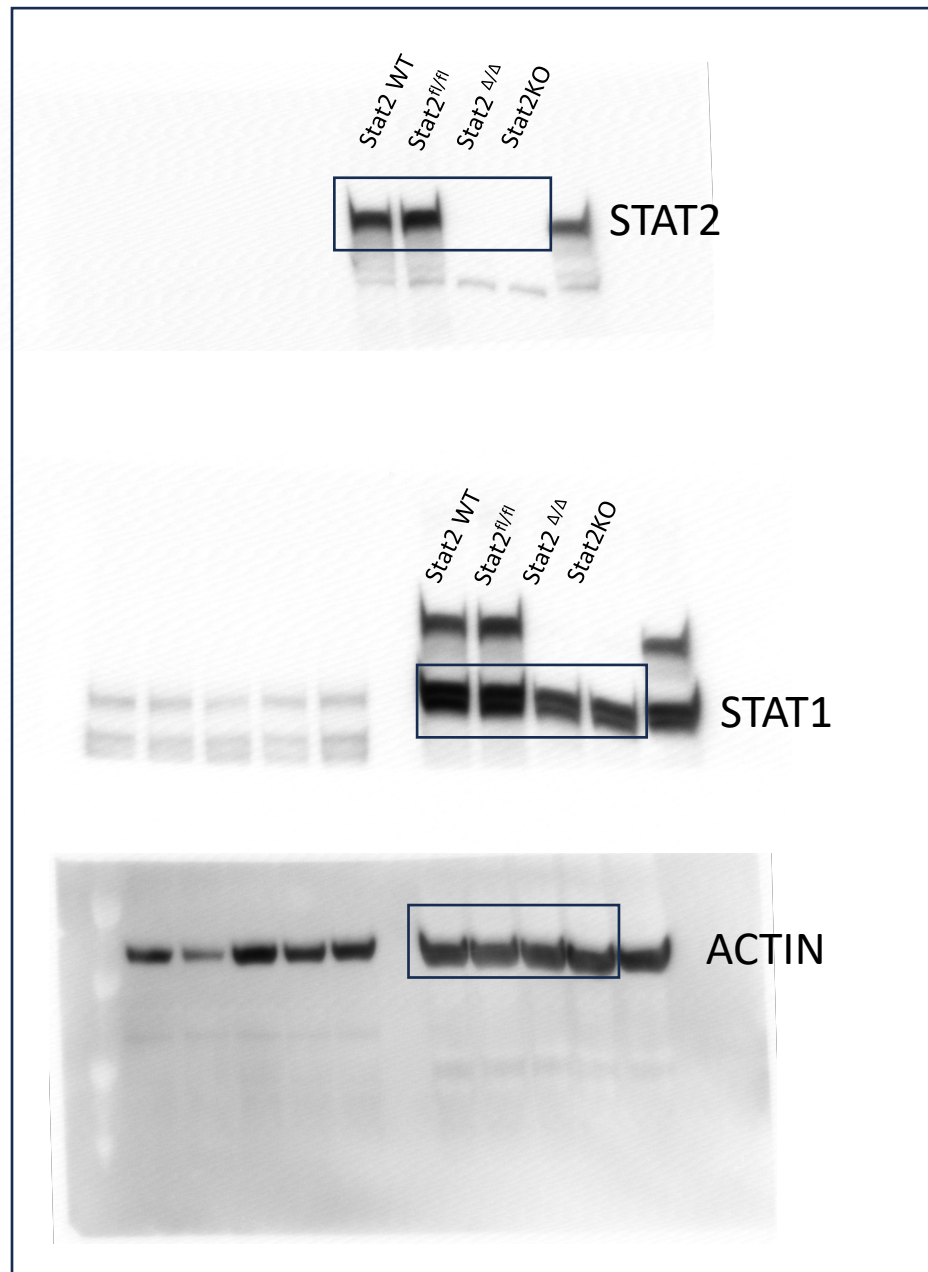

Figure S3 (Figure 4b)

Lung fibroblasts

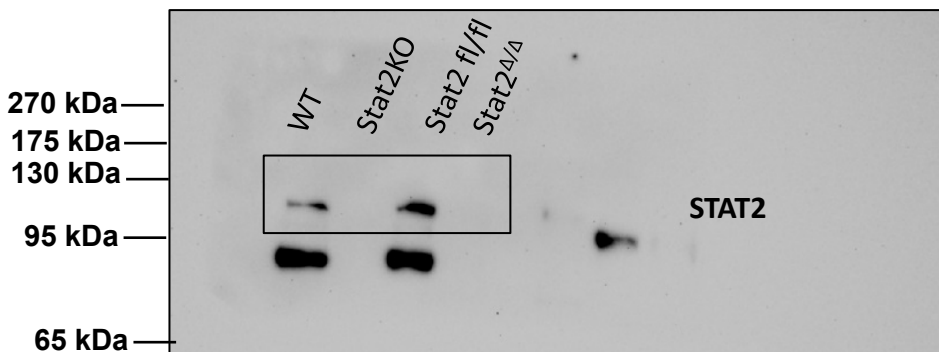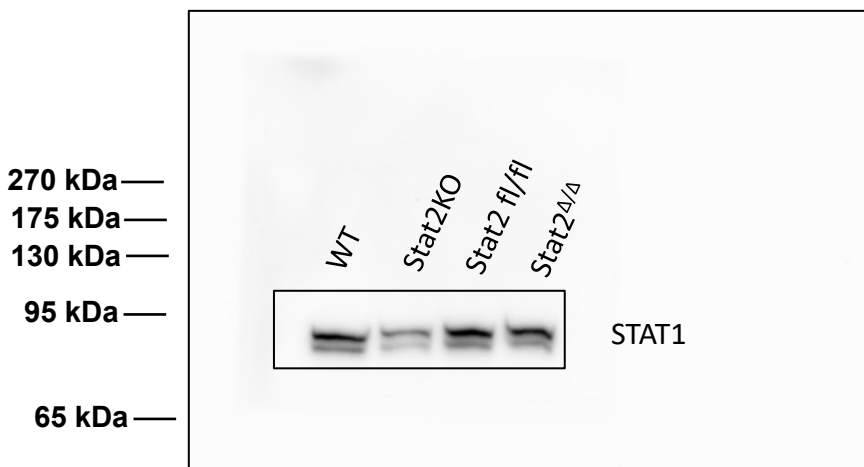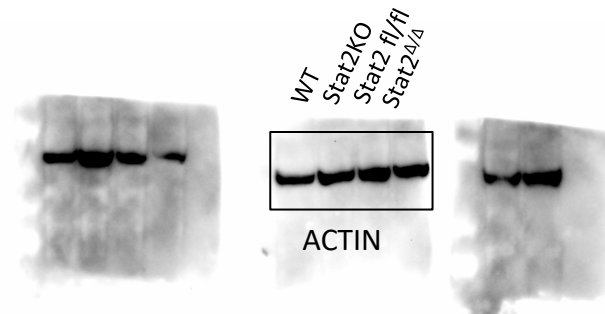

Figure S4 (Figure 5)

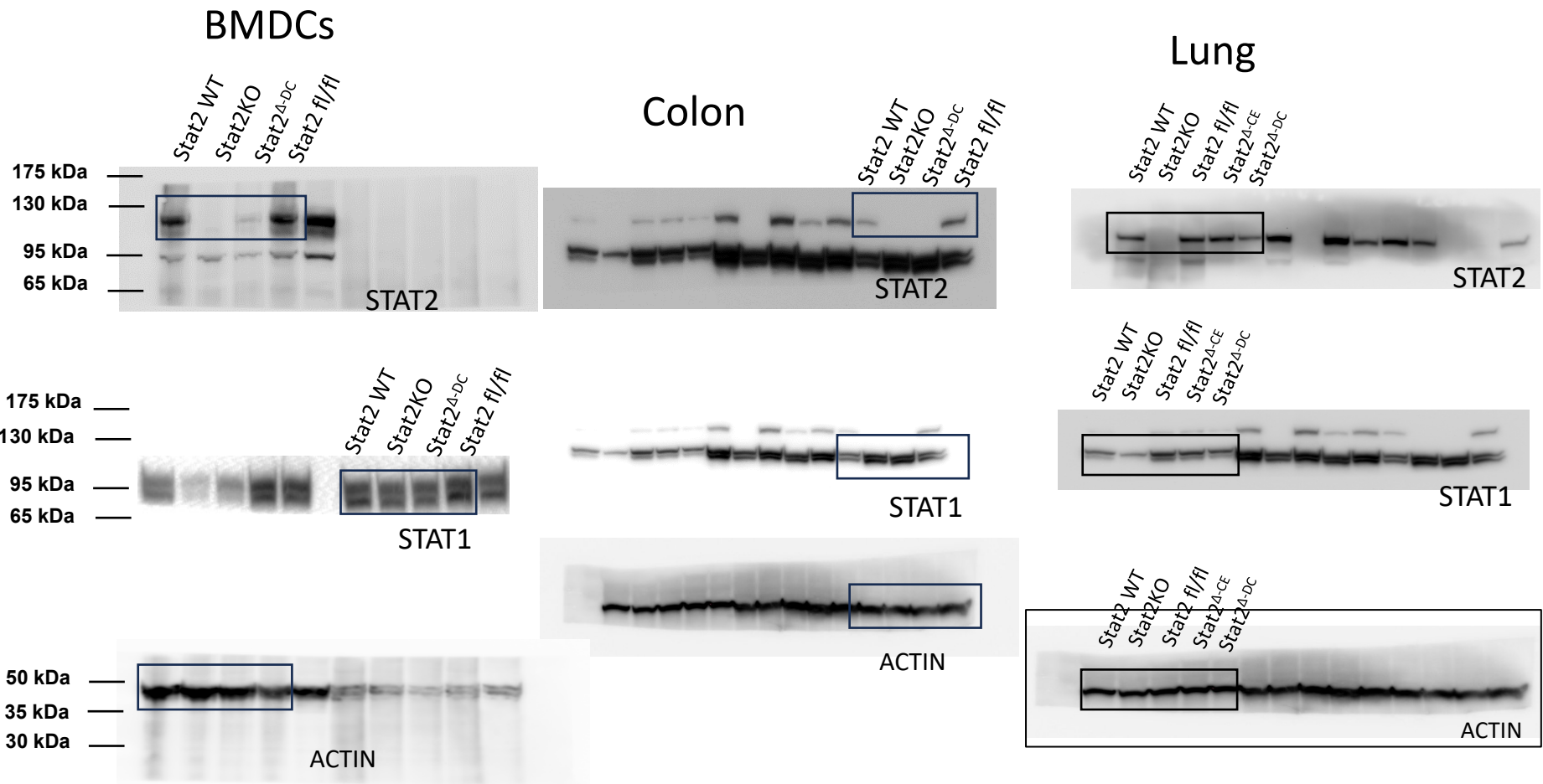

Supplement: supplementary materials [file NIHMS2151615-supplement-supplementary_materials.pdf]
